# First Report of Outbreaks of the Fall Armyworm *Spodoptera frugiperda* (J E Smith) (Lepidoptera, Noctuidae), a New Alien Invasive Pest in West and Central Africa

**DOI:** 10.1371/journal.pone.0165632

**Published:** 2016-10-27

**Authors:** Georg Goergen, P. Lava Kumar, Sagnia B. Sankung, Abou Togola, Manuele Tamò

**Affiliations:** 1 International Institute of Tropical Agriculture (IITA), 08 BP 0932 Tri Postal, Cotonou, Benin; 2 IITA, PMB 5320, Ibadan, Nigeria; 3 FAO, BP 2643, Libreville, Gabon; 4 IITA, PMB 3112, Kano, Nigeria; Pennsylvania State University, UNITED STATES

## Abstract

The fall armyworm *Spodoptera frugiperda* is a prime noctuid pest of maize on the American continents where it has remained confined despite occasional interceptions by European quarantine services in recent years. The pest has currently become a new invasive species in West and Central Africa where outbreaks were recorded for the first time in early 2016. The presence of at least two distinct haplotypes within samples collected on maize in Nigeria and São Tomé suggests multiple introductions into the African continent. Implications of this new threat to the maize crop in tropical Africa are briefly discussed.

## Introduction

Large numbers of larval armyworms plaguing various crops of economic importance are recurrently recorded in many countries of tropical Africa [1]. Severe outbreaks usually coincide with the onset of the wet season, especially when the new cropping season follows a long period of drought. When first observations of armyworms were made in late January 2016 on maize plants in the rainforest zone of South-Western Nigeria and in maize fields at the International Institute of Tropical Agriculture (IITA) at Ibadan and Ikenne, attacks were initially attributed to indigenous species of the genus *Spodoptera* Guenée, 1852 (Lepidoptera: Noctuidae) commonly occurring in West Africa. However, later in the season, further complaints about high armyworm populations arrived from northern Nigeria, Benin and Togo. In April 2016, following distress calls by maize producers, the government of São Tomé and Príncipe called for assistance from FAO through its sub-regional office for Central Africa (SFC) who expedited a technical mission to the country to assess the situation. Similarly by early June 2016, the Federal Government of Nigeria raised alarm over the upsurges of armyworm on maize in Edo and some adjacent states in the south west of the country. Evidently, these congruent reports from several West and Central African countries about sudden and severe outbreaks of armyworm populations pointed to the possible emergence of a new regional problem.

## Materials and Methods

### Ethics statement

All examined samples were collected on agricultural land and hence no specific permits were required for their collection in any of the referred locations. Pest management activities are an integral part of the research mandate contracted by the International Institute of Tropical Agriculture (IITA), a non-profit making international organisation and a member of the Consultative Group on International Agricultural Research Consortium (http://www.iita.org/). IITA is deeply implicated in research-for-development work carried out on the basis of formalized collaboration with national partners of 25 countries in the humid and sub-humid zones of sub-Saharan Africa. Samples from Togo, São Tomé & Príncipe and partially from Nigeria were submitted to IITA for accurate identification in the framework of a free regional identification service. The species presently studied is an agricultural pest and thus it is not recorded in any list as endangered or protected species.

### Sample collection

**Fig 1:**
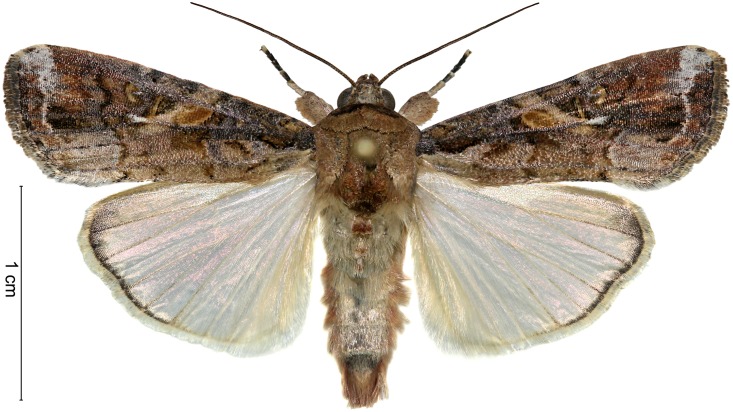
Adult male of *S*. *frugiperda* (Benin, Calavi Akassato, 6.vi.2016, G. Goergen). Photo by GG.

In addition to own collection efforts, samples from several sources in West and Central Africa were sent for accurate diagnostic to the IITA station in Cotonou, Benin, where morphological characters of immature stages and adult moths, including male and female genitalia, were examined using keys [2–3]. This led to a positive identification of the fall armyworm *Spodoptera frugiperda* (J.E. Smith) (Lepidoptera: Noctuidae), a species native to the tropical and subtropical regions of the Americas (Fig 1). Presently specimens collected on maize from the following locations constitute the first evidence for the occurrence of the fall armyworm on the African continent: **BENIN**: Calavi Akassato, 6°29'53.41"N, 2°21'46.08"E, 2.vi.2016, coll. G. Goergen; Allada, 6°39'39.02"N, 2°9'40.93"E, 2.vi.2016, coll. G. Goergen; Sékou, 6°38'11.54"N, 2°13'5.27"E, 2.vi.2016, coll. G. Goergen; Zé, 6°35'24.036"N, 2°16'37.74"E, 2.vi.2016, coll. G. Goergen; Glo, 6°33'40.50"N, 2°17'30.12"E, 2.vi.2016, coll. G. Goergen; Bohicon, 10 km south, 7°5'10.01"N, 2°6'16.06"E, 15.vi.2016, coll. O. Ajuonu; Massi, 7°0'21.11"N, 2°12'26.08"E, 15.vi.2016, coll. O. Ajuonu; **NIGERIA**: Ibadan, Oyo state, 7°26'5.3"N, 3°53'14.5"E, iii.2016, coll. O. Odeyemi; Ibadan IITA, Oyo state, 7°30'10.57"N, 3°53'47.59"E, iii.2016, coll. A. Togola & R. Aderanti; Ikenne, Ogun State, 6°50'58.8"N, 3°42'20.6"E, iii.2016, coll. O. Odeyemi; Kadawa, Kano state, 11°38'33"N, 8°25'58"E, 10.vi.2016, coll. A. Togola; Kano state, Gidan Ala, 11°36'9"N, 8°27'38"E, 10.vi.2016, coll. A. Togola; **SÃO TOMÉ and PRINCÍPE**: Porto Allegre, 0°2'6.39"N, 6°31'53.47"E, 26.iv.2016, coll. L. er Joro; Mesquita, 0°20'41.90"N, 6°41'50.84"E, 25.iv.2016, coll. L. er Joro; Pinheira, 0°17'7.20"N, 6°42'58.29"E, 26.iv.2016, coll. L. er Joro; **TOGO**: Lomé, University of Lomé, 6°10'33.72" N, 1°12'38.28" E, 14.vi.2016, coll. K. Agboka.

### Molecular analysis

To further confirm the species identity, larval and adult specimens from Nigeria and São Tomé & Príncipe were analyzed by ‘DNA barcoding’ at the Virology and Molecular Diagnostics Unit at the IITA Headquarters in Ibadan, Nigeria. The standard protocols were applied for DNA extraction, amplification of ~650 bp DNA portion of the mitochondrial cytochrome oxidase subunit I (COI) encoding gene commonly used for ‘DNA barcode’ analysis using the primer pair, LepF1 (5′- ATTC AACC AATC ATAA AGAT ATTGG -3′ and LepR1 (5′-TAAA CTTC TGGA TGT CCAA AAA ATCA-3′) [4]. Amplified products were purified and sequenced in both orientations, and the sequences (633 bp) were deposited in the NCBI GenBank.

**Fig 2:**
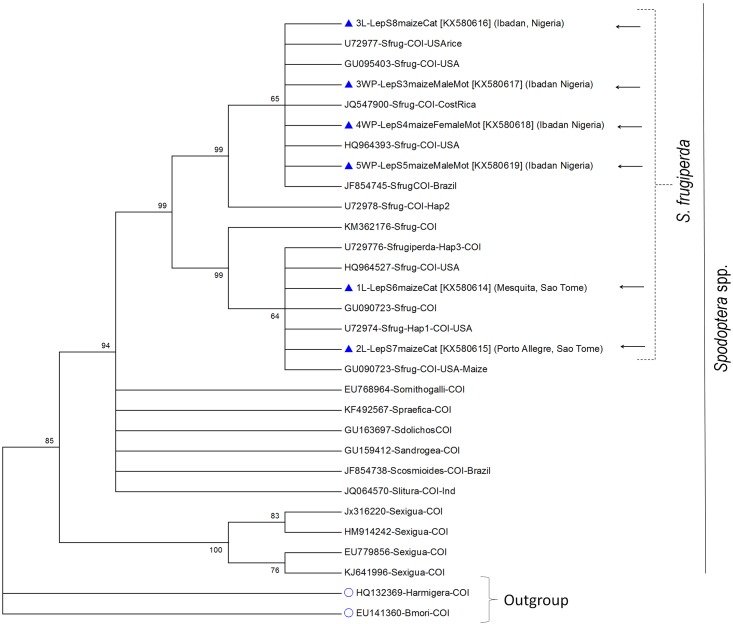
The phylogenetic relationships of 6 samples, 4 from Nigeria and 2 from São Tomé & Príncipe (arrows), inferred from the 633 bp mitochondrial cytochrome oxidase I (COI) sequence using the Maximum Likelihood method based on the Tamura-Nei model [20]. Bootstrap values (1,000 replications) reproduced in <60% replicates were collapsed and the bootstrap values are shown at the branch points. The GenBank accession numbers of the corresponding reference sequence are indicated. The analysis involved 30 nucleotide sequences and there were a total of 603 positions in the final dataset. Initial tree(s) for the heuristic search were obtained automatically by applying Neighbor-Join and BioNJ algorithms to a matrix of pairwise distances estimated using the Maximum Composite Likelihood (MCL) approach, and then selecting the topology with superior log likelihood value. The tree is drawn to scale, with branch lengths measured in the number of substitutions per site. Codon positions included were 1st+2nd+3rd+Noncoding. All positions containing gaps and missing data were eliminated. The nucleotide sequences were aligned using the MUSCLE [21] and the evolutionary analyses were conducted in MEGA6.06 [20].

Extractions of following material were submitted to a homology test using BLAST: **NIGERIA**: Ibadan, iii.2016, coll. A. Togola & R. Aderanti, 1 adult ♂, 1 adult ♀ & 1 immature; **SÃO TOMÉ and PRINCÍPE:** Porto Allegre, 26.iv.2016, coll. L. er Joro, 1 adult ♂ and 1 adult ♀; Mesquita, 25.iv.2016, coll. L. er Joro, 1 immature. The CO-I sequences presently generated were deposited in GenBank under accessions Nos. KX580614 to KX580619 (Fig 2). Voucher samples of adult moths were retained in the reference collection of the Biodiversity Centre at the IITA station in Benin.

## Results and Discussion

The DNA sequences of specimens from each country were 100% identical to each other, and they were 98% identical compared to specimens between the countries. A BLASTn search of DNA barcodes from Nigerian specimens (BankIt # 1937482, 1937497) revealed 100% nucleotide sequence identity with *S*. *frugiperda* voucher specimens with GenBank accessions Nos. HQ964393, GU095403, U72977; and those from the São Tomé & Príncipe (BankIt # 1937461) also showed 100% sequence identity with *S*. *frugiperda* but with a different set of voucher specimens (GenBank accessions Nos. GU090723, U72975, U72977). Further analysis of the DNA barcodes generated in this study along with the representative specimens of *S*. *frugiperda* and other *Spodoptera* spp., available in the NCBI GenBank with the Maximum-Likelihood phylogenetic analysis using the MEGA 6.06 program [5] aligned specimens from each country to a separate sub-cluster on *S*. *frugiperda* clade (Fig 2). This data ascertained the species identity established using morphological analysis and also demonstrated diversity between the specimens from the two countries indicating possibility of separate events of introduction.

The genus *Spodoptera* encompasses 31 species distributed on six continents [2]. Of the seven species previously recorded from the Afrotropical region, all except *S*. *malagasy* Viette, are known to occur in West and Central Africa namely *S*. *cilium* Guenée, *S*. *exempta* (Walker), *S*. *exigua* (Hübner), *S*. *littoralis* (Boisduval), *S*. *mauritia* (Boisduval) and *S*. *triturata* (Walker) [2,6]. External larval characteristics of *S*. *frugiperda* may casually lead to confusion with caterpillars of *S*. *exigua*, the beet armyworm, which—until now—is the only known cosmopolitan species within the genus *Spodoptera*. The latter species can considerably vary in colour and tegument pattern [7]. In outbreak areas, it is regularly encountered together with *S*. *exempta*, the African armyworm. With its new introduction into the African continent, *S*. *frugiperda* extents considerably its aboriginal range, the tropical regions of the Americas from the United States to Argentina and the Caribbean region [2].

The fall armyworm is a voracious pest and, given its polyphagous nature, it is expected that its accidental introduction in the African continent will constitute a lasting threat to several important crops. It has a wide host range with almost 100 recorded plant species in 27 families [2,8]. The preferred hosts are graminaceous plants, including economically important crops such as maize, millet, sorghum, rice, wheat, and sugar cane. Feeding damage is also observed on other major agricultural crops such as cowpea, groundnut, potato, soybean and cotton.

**Fig 3:**
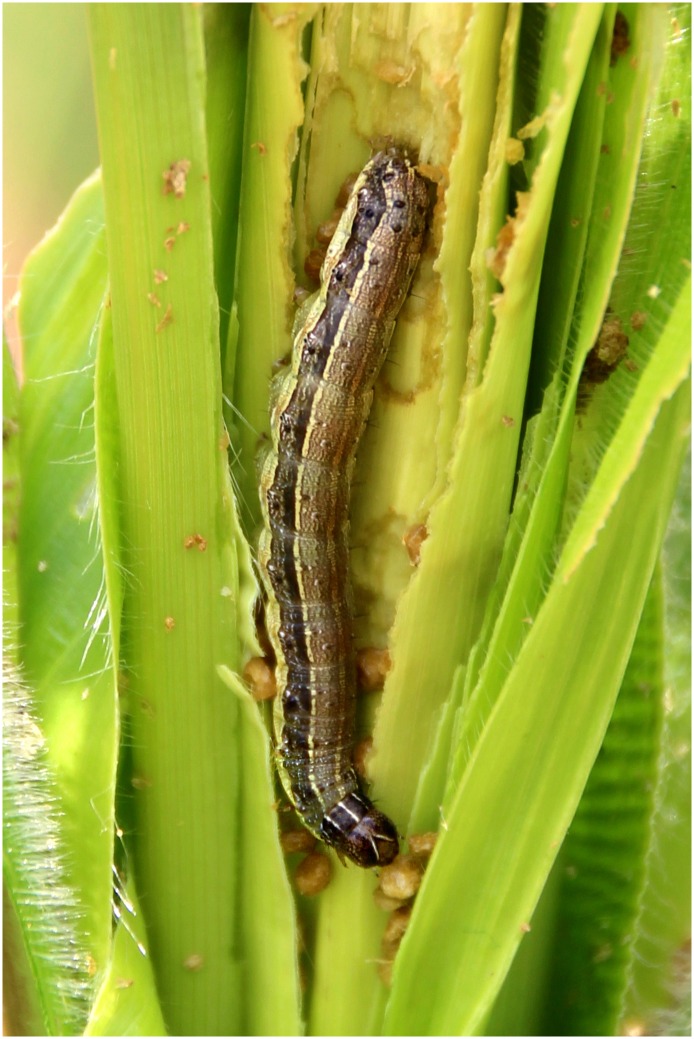
Fifth instar larva of *S*. *frugiperda* (Benin, Calavi Akassato, 2.vi.2016, G. Goergen). Photo by GG.

Several factors suggest that *S*. *frugiperda* is likely to become more damaging to maize than other species of the same genus occurring in Africa: a) Whereas congeneric afrotropical armyworms first build up dense populations on wild grasses before older larvae move onto cultivated graminaceous crops [1], adult females of *S*. *frugiperda* directly oviposit on maize. b) Unlike in most other *Spodoptera* species in Africa, the mandibles of caterpillars of the fall armyworm have comparatively stronger, serrated cutting edges, which ease the feeding on plants with high silica content [2,6]. c) Older larvae (Fig 3) become cannibalistic and have the ability to dominate interspecific competitors and reduce intraspecific rivals [9]. d) As shown in several countries in the tropical Americas, where climatic conditions allow a constant reproduction of the pest, the damage inflicted to maize is particularly severe. Thus, *S*. *frugiperda* is considered the most important pest of maize in Brazil [10], the third largest maize producer in the world after the USA and China. For this country alone, costs to control the fall armyworm on maize exceed 600 million dollars annually [11].

The presence of this new pest in West and Central Africa adds to the threat already caused by native lepidopteran maize stalk/stem- or ear-borers of economic importance, in particular the Noctuidae *Busseola fusca* (Fuller), *Sesamia calamistis* Hampson, and the Pyralidae *Eldana saccharina* Walker and *Mussidia nigrivenella* (Ragonot) [12,13]. The economic consequences of the establishment of *S*. *frugiperda* on the African continent may not be limited to its direct effects on agricultural production but also has the potential to adversely affect access to foreign markets. In recent years, the rates of quarantine interceptions of fall armyworm caterpillars on fresh vegetables and living plants at European entry points have significantly increased [14]. As a result, the status of *S*. *frugiperda* was reassessed in 2015 and ranked as A1 quarantine pest on the list of the European and Mediterranean Plant Protection Organization [7]. With its new range extension, it is anticipated that the fall armyworm will soon be included in the list of quarantine pests of other regional plant protection organizations.

**Fig 4:**
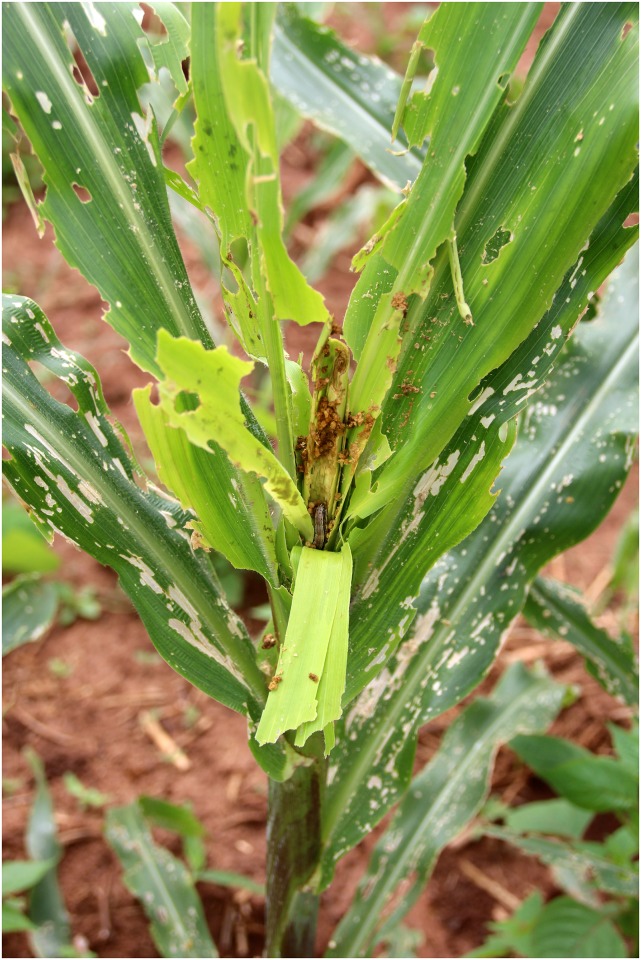
Adult male of *S*. *frugiperda* (Benin, Calavi Akassato, 6.vi.2016, G. Goergen). Photo by GG.

Damage on maize may be observed on all plant parts depending on development stage. Larger caterpillars act as cutworms by entirely sectioning the stem base of maize plantlets. During the maize vegetative phase, constant feeding results in skeletonized leaves and heavily windowed whorls loaded with larval frass (Fig 4). On grown maize plants, larvae also attack reproductive organs feeding on tassels or boring into the ears. Following hatching, neonate larvae usually bore into the host plant and develop under protected conditions. Hence control with contact insecticides is often ineffective though it remains until today the most widely practiced management measure. Its frequent overuse has led to the emergence of regional populations resistant to several classes of pesticides [15] and favoured the use of transgenic Bt-maize. Thus, cultivars expressing the Cry1F toxin against insect defoliators are currently widely commercialized in the western hemisphere. Owing to economic, logistic and socio-cultural considerations, such regular insecticide applications and the deployment of transgenic Bt-maize are not as straightforward in tropical Africa. Moreover, reports about increasing cases of fall armyworm resistance to Cry1F [16] underpin the need to develop alternative control options including the use of nucleopolyhedroviruses (NPV), endophytic entomopathogenic fungi and insect biological control agents.

Pathways of the introduction of fall armyworm into West and Central Africa are subject to speculations; but the presence of at least two distinct haplotypes within the collected material suggests that the present incursion originated from at least two introductions (Fig 2). *Spodoptera frugiperda* has a remarkable dispersal capacity, a feature that is understood to have evolved as part of its life history strategy [17]. Thus, in annual migrations, the pest is able to expand from its endemic area in the warmer parts of the New World over more than 2000 km across the entire US up to Canada in the North and reaching the northern parts of Argentina and Chile in the South [18]. How far *S*. *frugiperda* has already expanded onto the African continent remains to be determined; but considering its high spreading performance, large reproductive capacity [19], absence of diapause [17], and wide host plant range it is likely that the pest will soon be able to colonize most of tropical Africa. Hence, there is an urgent need for developing ecologically sustainable, economically profitable and socially acceptable IPM programs to mitigate the impact of the fall armyworm in Africa.
